# Computational modeling and minimization of unintended neuronal excitation in a LIFU stimulation

**DOI:** 10.1038/s41598-023-40522-w

**Published:** 2023-08-17

**Authors:** Boqiang Fan, Wayne Goodman, Raymond Y. Cho, Sameer A. Sheth, Richard R. Bouchard, Behnaam Aazhang

**Affiliations:** 1 Department of Electrical and Computer Engineering, Rice University, Houston, TX 77005 USA; 2 Department of Psychiatry and Behavioral Science, Baylor College of Medicine, Houston, TX 77030 USA; 3 Department of Neurosurgery, Baylor College of Medicine, Houston, TX 77030 USA; 4 Department of Imaging Physics, University of Texas MD Anderson Cancer Center, Houston, TX 77030 USA

**Keywords:** Biomedical engineering, Neuroscience, Acoustics

## Abstract

The neuromodulation effect of low-intensity focused ultrasound (LIFU) is highly target-specific. Unintended off-target neuronal excitation can be elicited when the beam focusing accuracy and resolution are limited, whereas the resulted side effect has not been evaluated quantitatively. There is also a lack of methods addressing the minimization of such side effects. Therefore, this work introduces a computational model of unintended neuronal excitation during LIFU neuromodulation, which evaluates the off-target activation area (OTAA) by integrating an ultrasound field model with the neuronal spiking model. In addition, a phased array beam focusing scheme called constrained optimal resolution beamforming (CORB) is proposed to minimize the off-target neuronal excitation area while ensuring effective stimulation in the target brain region. A lower bound of the OTAA is analytically approximated in a simplified homogeneous medium, which could guide the selection of transducer parameters such as aperture size and operating frequency. Simulations in a human head model using three transducer setups show that CORB markedly reduces the OTAA compared with two benchmark beam focusing methods. The high neuromodulation resolution demonstrates the capability of LIFU to effectively limit the side effects during neuromodulation, allowing future clinical applications such as treatment of neuropsychiatric disorders.

## Introduction

Low-intensity focused ultrasound (LIFU) neuromodulation, an emerging therapeutic tool for altering neuronal activity, has received increased attention for treating various neuropsychiatric disorders^1–3^. Compared with traditional stimulation techniques such as transcranial magnetic stimulation, LIFU has the potential to reach deep targets inside the brain and achieve reversible neuromodulation effects with millimeter-level focusing resolution while being markedly less invasive than deep brain stimulation^4^.

Recent studies have shown that the LIFU neuromodulation effect is sensitive to the stimulated region. Different target locations of ultrasound power focusing can result in different sensations and motor responses^5,6^, even with a separation of merely 1 mm. In addition, neuromodulatory effects are not limited to the target location only. Off-target focal pressure peaks can occur during stimulation due to standing waves, which potentially resulted in the reported neuromodulatory effects in many previous experiments^6,7^. Opposite neuromodulation effects can also occur at the same target using focused and unfocused ultrasound stimulation, due to differences in the number of neurons affected^8^.

Although such unintended neuromodulation effects can be potentially observed in many scenarios, there is still a lack of quantitative evaluations on the corresponding influence, i.e., the specific brain regions affected. Accordingly, the methods for minimizing the unintended neuronal excitation have also not been well investigated yet, even though the dependence of the ultrasound focal spot size on transducer design parameters has been understood^3,6,9^. Hence, to quantitatively evaluate the unintended neuronal excitation, this work proposes to computationally model the off-target activation area (OTAA) of LIFU neuromodulation, which indicates the region with unintended neuronal spiking responses to LIFU while the target is successfully excited simultaneously^6,10^. The computational modeling of OTAA is mainly based on the interaction between the ultrasound propagation model and the multi-scale optimized neuronal intramembrane cavitation (SONIC) model that allows quantitative interpretation of LIFU neuronal excitation effects^11^. To effectively minimize the OTAA, this work considers using phased array transducers for LIFU stimulation, which allows more flexible control of the ultrasound radiation pattern compared to the single-element transducers used in most literature. A new beam focusing algorithm, named constrained optimal resolution beamforming (CORB), is proposed to minimize unintended neuronal excitation effects on off-target brain regions while ensuring effective stimulation of the target point. The OTAA with CORB used is computationally evaluated in a 2-D human head model^12^ given three potential transducer deployments (transcranial/intracranial/double-array). Compared to traditional beam focusing algorithms, CORB method elicits much smaller OTAA while ensuring successful stimulation to the target neurons.

## Methods

### Computational modeling of unintended neuronal excitation

**Figure 1:**
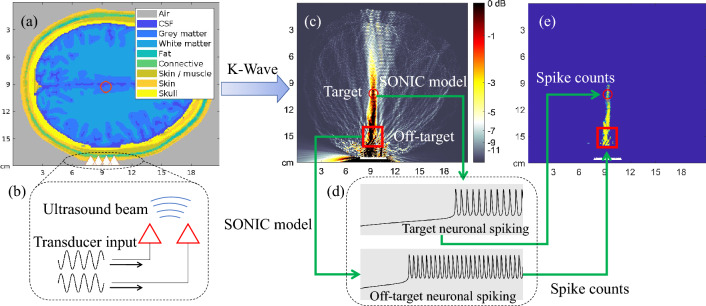
The computational modeling method of unintended neuronal excitation during LIFU stimulation. (**a**) A 2-D human head model^12^ with a target point denoted by the red circle and ultrasound transducer elements denoted by white triangles. (**b**) An illustrative figure of the input signals for phased array transducer elements to focus the beam. (**c**) The ultrasound intensity distribution in the human head model generated by the transducer beam, simulated with the k-Wave toolbox. (**d**) The elicited neuronal spiking patterns of on-target and off-target brain regions, simulated with SONIC model given the ultrasound waveform (500 kHz continuous wave), intensity (0.078 and 0.175 W/cm$^{2}$ for on-target and off-target regions), and the stimulation duration (100 ms), etc., of that region. (**e**) The map that demonstrates the number of neuronal spikes elicited in different brain regions. The highlighted region except the target point is defined as OTAA, showing the influence of unintended neuronal excitation.

This work computationally models the unintended neuronal excitation during LIFU neuromodulation relying on a unified simulation framework of ultrasound propagation and neuronal responses. The modeling methods are illustrated in Fig. 1. Given a human head model as in Fig. 1a and the ultrasound transducer inputs for beam focusing as in Fig. 1b, the ultrasound intensity distribution in brain tissues, as in Fig. 1c, can be simulated with the k-Wave toolbox^13^. The SONIC model can then be applied to calculate the spiking pattern of neurons in each brain region, as in Fig. 1d, using the exact ultrasound pressure of the beam in that region as the input^11^. To demonstrate the influence of unintended neuronal excitation, this work evaluates OTAA, i.e., the off-target area of neurons excited. The OTAA measures the highlighted area in Fig. 1e, which denotes the brain region with non-zero neuronal spikes elicited, given that the target neuron is concurrently excited.

**Figure 2:**
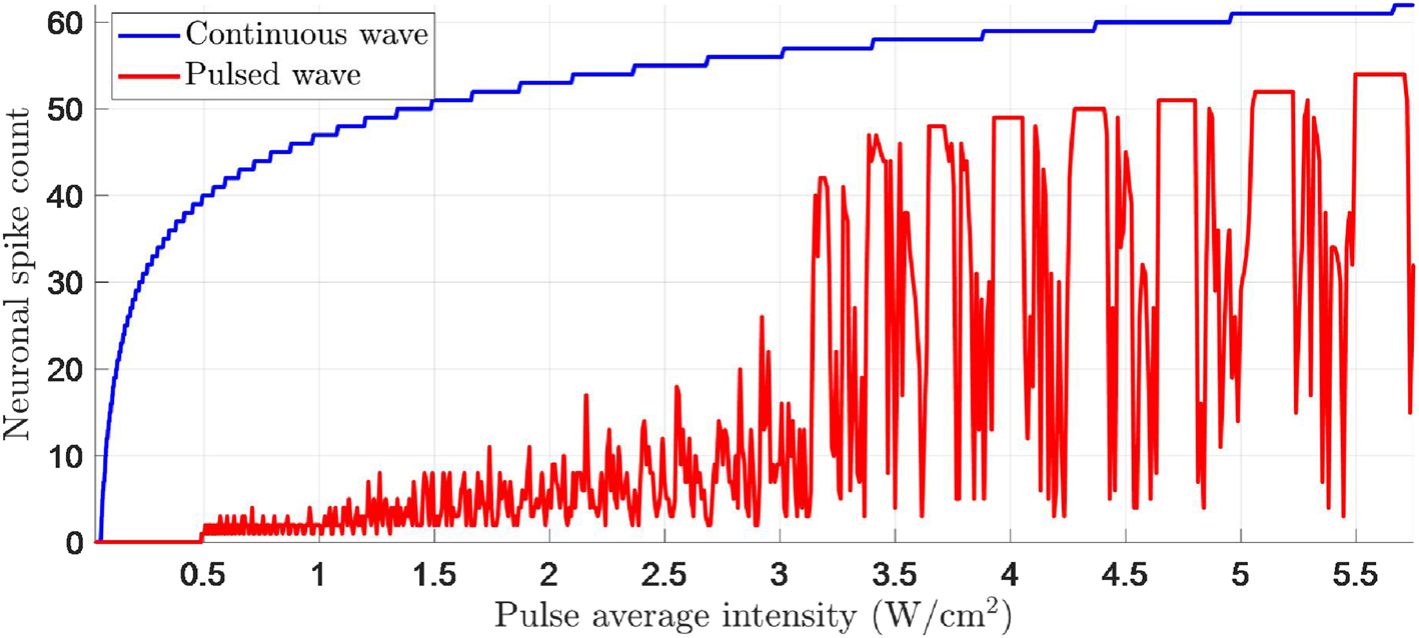
The number of neuronal spikes elicited by continuous-wave (duration: 100 ms) and pulsed-wave (duration: 100 ms; pulse repetition frequency (PRF): 1000 Hz; duty cycle: $36 \%$) stimulation simulated with the SONIC model. The dependence of neuronal spike counts on LIFU intensities during continuous-wave and pulsed-wave stimulations is demonstrated.

The SONIC model can simulate excitatory neuronal spiking responses to ultrasound stimulation, which rely on several factors, including the type of neuron, stimulation signal parameters (e.g., pressure, duration, duty cycle, ...), etc. In the current study, the spiking pattern of regular spiking neurons is modeled using SONIC, the analysis of which can be extended to various types of neurons in future research. Other factors, including brain region and anesthesia state, are beyond the scope of the current study. Simulated neuronal spiking patterns under continuous and pulsed LIFU stimulation using different ultrasound intensities are shown in Fig. 2. The numbers of neuronal spikes elicited by LIFU stimulation under different ultrasound intensities are demonstrated, given other parameters for continuous-wave and pulsed-wave stimulation, respectively, are fixed. Neuronal excitation intensity thresholds for both continuous waves (e.g., $\sim 0.05$ W/cm$^{2}$) and pulsed waves (e.g., $\sim 0.45$ W/cm$^{2}$) are observed, aligning with experimental results and theories in which minimum intensity thresholds for successful ultrasound neuronal excitation exist^11,14–18^. In addition, although the neuronal spike count generally increases with intensity in both the continuous-wave and pulsed-wave scenarios, such an increase with pulsed waves of low duty cycles is slower and non-monotonic, which could influence the neuronal excitation resolution as discussed in further numerical evaluations.

### Beam focusing model

This work evaluates the unintended neuronal excitation elicited with three beam focusing methods, including two benchmark methods, i.e., the conjugate beamforming benchmark and the off-target suppression benchmark, and the proposed CORB method. The conjugate benchmark maximizes ultrasound intensity at the target point, which has similar mechanism to most focusing methods used in literature, e.g., time-reversal method^19^. The off-target suppression benchmark maximizes the power focused to the target point while simultaneously limiting the power delivered to all off-target brain regions^20^. The details of these benchmark methods are introduced in the Supplementary Information.

#### Modeling ultrasound propagation and intensity for CORB

The CORB method optimizes transducer array beam focusing relying on the ultrasound propagation and intensity model in tissues, which is described as follows. It is assumed that an *M*-element transducer array operating at frequency *f* is used for neuromodulation. An input sine signal with constant amplitude $A_{m}$ and phase $\phi _{m}$ continuously drives each transducer element *m*, generating the acoustic field during a pulse with duration *T* or a continuous wave stimulation^16,17^. The acoustic pressure generated by the waveform of each single element *m* is denoted by $p_{m}(G,t)$ at any point *G* in the brain tissue region. If the wave equation in k-Wave^13^ is considered linear, $p_{m}(G,t)$ is the linear superposition of all incident and reflected (e.g., those from the skull) sine waves, which will become a regular sine wave after transients have died out. The complex-domain representation of $p_{m}(G,t)$ is denoted by $\tilde{p}_{m}(G,t)$, which can be acquired using the Hilbert transform,

1 $$\begin{aligned} \begin{aligned} \tilde{p}_{m}(G,t)&= A_{m} h_{m}(G,t) e^{j(2\pi f t + \phi _{m} - \frac{\pi }{2})}, \end{aligned} \end{aligned}$$

where $h_{m}(G,t)$ represents the total amplitude and phase changes from the element *m* to the point *G*. The cumulative acoustic signal at a given point can be modeled as a linear combination of signals from all active transducer elements, i.e., $\tilde{p}(G,t) = [\varvec{h}(G,t)]^{H} \varvec{w} e^{j(2\pi f t - \frac{\pi }{2})}$. Here $\varvec{w} = [w_{1},\cdots ,w_{M}]^{H}$ is the beam focusing vector for the phased-array transducer and $w_{m} = A_{m} e^{-j\phi _{m}}$ for any array element *m*, where $[\cdot ]^{H}$ represents the conjugate transpose operator. The matrix $\varvec{h}(G,t) = [h_{1}(G,t),\cdots ,h_{M}(G,t)]^{H}$ models the propagation from the phased array transducer to *G*. Therefore, the average ultrasound intensity at any point *G*, which is used in CORB to calculate neuronal spiking responses to LIFU stimulation, is^21,22^

2 $$\begin{aligned} I(G) = \frac{ [\tilde{p}(G,t)]^{H} \tilde{p}(G,t) }{2\rho (G)c(G)} = [\varvec{w}]^{H} \varvec{Q}(G) \varvec{w}, ~\varvec{Q}(G) = \frac{ \varvec{h}(G,t) [\varvec{h}(G,t)]^{H} }{2\rho (G)c(G)}, \end{aligned}$$

where $\varvec{Q}(G)$ is $M \times M$. The ambient medium density and sound speed at point *G* are represented by $\rho (G)$ and *c*(*G*) , respectively.

#### Constrained optimal resolution beamforming (CORB)

The CORB method is designed to estimate neuronal spiking responses based on the ultrasound intensity model in the previous section, and minimize the unintended neuronal excitation area, which is subject to the excitation thresholds as illustrated in Fig. 2. It is assumed that the intensity at the target point $G^{*}$ must be higher than or equal to an excitation threshold $I_{h}$ to elicit sufficient neuronal spikes for successful stimulation; for any point *G* in the off-target brain region, the intensity needs to be smaller than a relatively “conservative” threshold $I_{l} \le I_{h}$ to avoid off-target neuromodulation effects. A “transition interval” between $I_{l}$ and $I_{h}$ can also improve the robustness to counter the probability and individual specificity in LIFU neuromodulation^17,18^. The values of $I_{l}$ and $I_{h}$ depend on the application.

Given the neuronal excitation thresholds, the unintended neuronal excitation can be modeled by OTAA, which can further be approximated by $S(\varvec{w})$ that estimates the insonification areas of off-target brain tissue with intensity *I*(*G*) higher than the “conservative” threshold $I_{l}$,

3 $$ \begin{aligned}{}&S(\varvec{w}) = \sum _{G \ne G^{*} } \chi \Big ( I(G) - I_{l} \Big ), ~\text {s.t.} ~~ I(G^{*}) \ge I_{h}.  \end{aligned}$$

The intensity at the target point $G^{*}$ needs to be ensured to exceed the excitation threshold $I_{h}$. The function $\chi (z)$ is a step function, implying $\chi (z) = 1$ for any real scalar $z \ge 0$ and otherwise $\chi (z) = 0$. In (3) the area is equivalently measured with the number of spatial points in the brain tissue region. Ideally, $S(\varvec{w}) = 0$ implies no off-target excitation and optimal neuromodulation resolution.

The goal of the CORB method is to achieve the minimal OTAA by minimizing $S(\varvec{w})$. The Supplementary Information shows that the optimal solution of the following formulation is an equivalent scaling of the optimal solution that minimizes (3),

4 $$\begin{aligned} &\underset{\begin{array}{c} \varvec{w} \in \mathbb {C}^{M} \end{array}}{\min }{} & {} \sum _{G \ne G^{*} } \chi \Bigg ( [\varvec{w}]^{H} \varvec{Q}^{*} (G) \varvec{w} \Bigg ), ~\text {s.t.} ~~ ||\varvec{w}||_{2}^{2} = P, ~ \varvec{Q}^{*} (G) = \varvec{Q}(G) - \frac{I_{l}}{I_{h}}\varvec{Q}(G^{*}). \\ \end{aligned}$$

The objective function minimizes the off-target area at an intensity higher than $\frac{I_{l}}{I_{h}}$ times the intensity at the target point $G^{*}$. The constraint requires the sum power output of the transducer array to be constant and serves for regularization. Here, *P* is defined as a scaling of the sum power of the transducer array, and $P = 1$ is assumed without loss of generality. The problem formulation in (4) represents a discontinuous non-convex constrained optimization problem that cannot necessarily be solved to reach the global optimum within a reasonable computational time using general optimization methods^23^. To solve this problem, the step function is first approximated using a logistic function, which yields the approximated objective function

5 $$\begin{aligned}&\underset{\begin{array}{c} \varvec{w} \in \mathbb {C}^{M} \end{array}}{\min }{} & {} \sum _{G \ne G^{*} } \Big (1 + e^{-\mu [\varvec{w}]^{H} \varvec{Q}^{*} (G) \varvec{w} } \Big )^{-1},  \end{aligned}$$

where the parameter $\mu$ that determines approximation accuracy is discussed in the Supplementary Information. A sub-optimal solution is sought for the approximated optimization problem (5) using the gradient projection method^24^. Because $\varvec{Q}^{*} (G)$ is a Hermitian matrix for any point *G* according to (2) and (4), the gradient of (5) with respect to the beamforming coefficient vector $\varvec{w}$ is $\mu \varvec{D}_{\mu } \varvec{w}$^25^, where $\varvec{D}_{\mu }$ is

6 $$\begin{aligned} \begin{aligned} \varvec{D}_{\mu } = \sum _{G \ne G^{*}} \frac{ e^{-\mu [\varvec{w}]^{H} \varvec{Q}^{*} (G) \varvec{w} } \varvec{Q}^{*} (G) }{ (1 + e^{-\mu [\varvec{w}]^{H} \varvec{Q}^{*} (G) \varvec{w} })^{2} }. \end{aligned} \end{aligned}$$

According to the gradient projection method, $\varvec{w}$ can be solved by iteratively updating $\varvec{w}^{(n+1)} = \mathbb {P} (\varvec{w}^{(n)} - \gamma \mu \varvec{D}_{\mu } \varvec{w}^{(n)})$, where $\varvec{w}^{(n)}$ denotes the beamforming vector updated after the *n*th iteration. The projection operator $\mathbb {P}(\cdot )$ normalizes the input vector to satisfy the transducer power constraint. Here $\gamma$ defines the step size. The initialization is set to $\varvec{w}^{(0)} = \varvec{w}_{conj}$ as defined in the Supplementary Information^19^. The iteration ends if $||\varvec{w}^{(n+1)} - \varvec{w}^{(n)}||_{2}$ is smaller than a threshold $\beta$. The transducer array using optimized $\varvec{w}$ as the input can generate LIFU beam patterns for neuronal excitation.

## Theoretical lower bound of OTAA

Even with optimized beam patterns, the OTAA can be substantially limited by transducer parameters, including the number of array elements and operating frequency. Therefore, it is important to approximate the minimal achievable area of unintended neuronal excitation given transducer parameters, so that the potential side effect can be estimated for clinical applications. Traditional theoretical results on focal sizes do not account for neuromodulation effects and are thus not suitable for OTAA analysis. To resolve this issue, the current study analytically derives a lower bound $S_{LB} \le S(\varvec{w})$ in a simplified homogeneous medium, which approximates the smallest OTAA that may not even be feasible. The detailed formula and derivation of the lower bound is discussed in the Supplementary Information.

## Numerical evaluation

### Simulation setups

**Figure 3:**
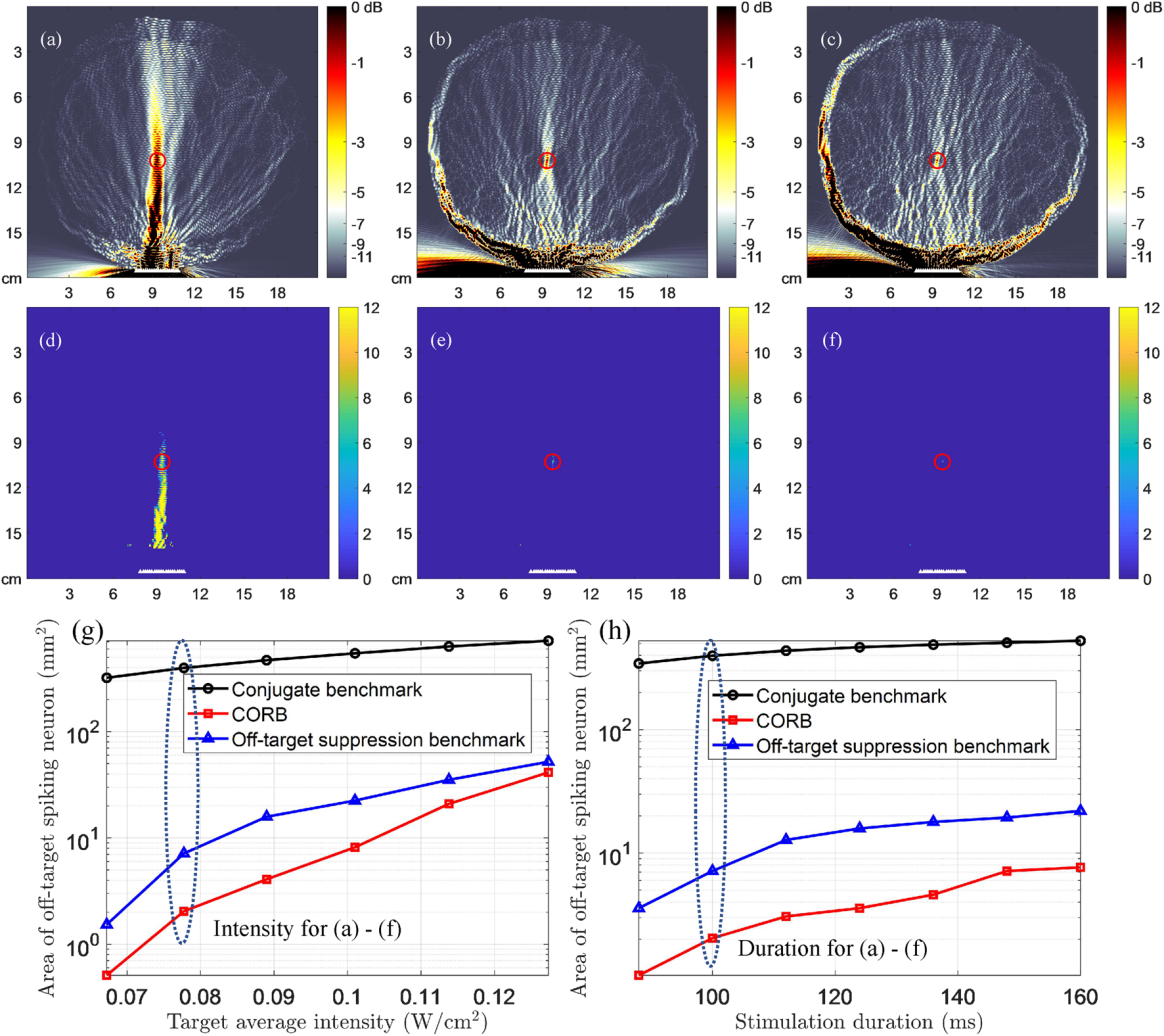
The beam patterns and neuronal spike patterns of transcranial stimulation with single transducer array, demonstrating the minimized OTAA of CORB compared with two benchmarks. The distance between the target point and array is approximately 7.33 cm. (**a**–**c**): Approximate beam patterns for continuous-wave stimulation generated by the conjugate benchmark, the off-target suppression benchmark, and CORB, respectively. The color maps demonstrate the spatial distribution of mean ultrasound intensity normalized by the target signal intensity (0.078 W/cm$^{2}$)^22^. (**d**–**f**): Neuronal spike count map of conjugate benchmark, the off-target suppression benchmark, and CORB, respectively, in a continuous-wave LIFU stimulation (target intensity: 0.078 W/cm$^{2}$; duration: 100 ms). Ultrasound pressures at all other points are scaled proportionally to the target based on the mean intensity distributions in (**a**)–(**c**). (**g**): The OTAA in log-scale with different stimulation intensities (duration: 100 ms). (textbfh) The OTAA in log-scale with different stimulation durations (target intensity: 0.078 W/cm$^{2}$).

The current study numerically evaluates the unintended neuronal excitation elicited by the three beam focusing methods in a 2-D cross-section of a human head model in Fig. 1a^12^. Three different transducer array placements are considered for evaluation as shown in Fig. S-1. The first placement involves a single array attached to the skin for transcranial stimulation. The second placement uses a single array for intracranial stimulation, in which an implanted array is attached to the inner surface of the skull on the dura. Such an array can be mounted using customized cranial implants that partially replace the skull^26^. The third placement uses a double-array system for intracranial stimulation, in which the arrays are attached at two locations on the inner surface of the skull and operate synchronously at the same frequency. Both continuous-wave and pulsed-wave stimulations are considered in the evaluation. The simulation parameters are mainly discussed in the Supplementary Information.

### Minimizing unintended neuronal excitation effects using the CORB method

Fig. 3a–c present the single-array transcranial beam patterns generated using two benchmark methods and CORB. Fig. 3d–f show maps of neuronal spike counts elicited by the ultrasound beam profiles of the three beamforming methods in Fig. 3a–c, resulting from the SONIC model. The colorbar in Fig. 3a–c denotes the relative ultrasound intensity to the target intensity, and in Fig. 3d–f denotes the number of spikes elicited at each point. Comparison shows that the conjugate benchmark provides the largest OTAA in Fig. 3d due to unconstrained effects in off-target regions. In contrast, the OTAA of the off-target suppression benchmark is markedly reduced to about 14.9 mm$^{2}$ in Fig. 3e, implying improved neuromodulation resolution. The CORB method further reduces the OTAA to about 3.1 mm$^{2}$ in Fig. 3f, which is a $79.2\%$ improvement compared with the off-target suppression benchmark. It is hence demonstrated that CORB can induce neuronal spiking with high spatial resolution and mitigate potential side effects.

**Figure 4:**
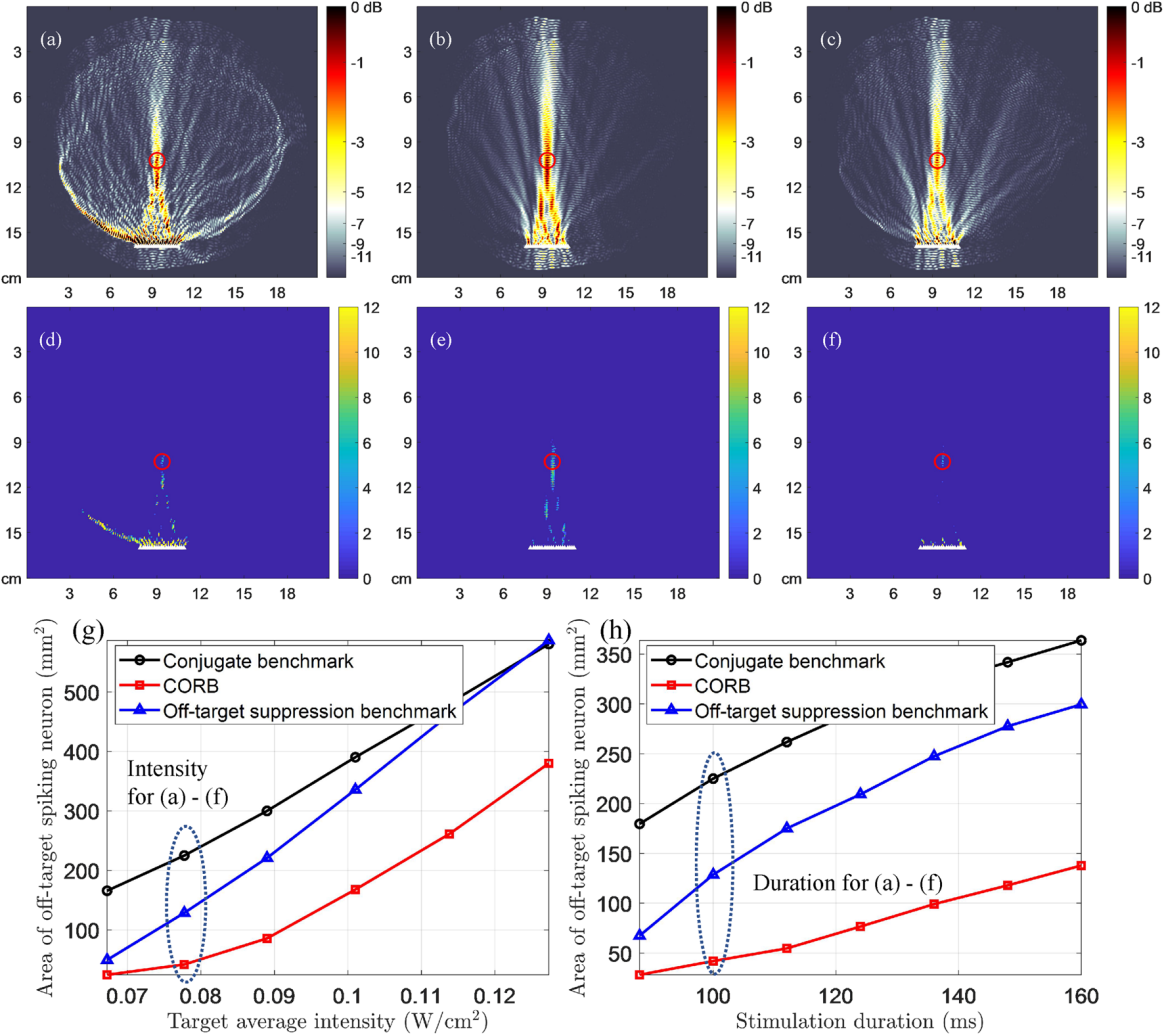
The beam patterns and neuronal spike patterns of intracranial stimulation with single transducer array, demonstrating the minimized OTAA of CORB compared with two benchmarks. The location of the target is the same as in Fig. 3, whereas the distance between the target point and array is approximately 5.71 cm. (**a**–**c**) Approximate beam patterns for continuous-wave stimulation generated by the conjugate benchmark, the off-target suppression benchmark, and CORB, respectively. The color maps demonstrate the spatial distribution of mean ultrasound intensity normalized by the target signal intensity (0.078 W/cm$^{2}$)^22^. (**d**–**f**) Neuronal spike count map of conjugate benchmark, the off-target suppression benchmark, and CORB, respectively, in a continuous-wave LIFU stimulation (target intensity: 0.078 W/cm$^{2}$; duration: 100 ms). Ultrasound pressures at all other points are scaled proportionally to the target based on the mean intensity distributions in (**a**–**c**). (**g**) The OTAA with different stimulation intensities (duration: 100 ms). (**h**) The OTAA with different stimulation durations (target intensity: 0.078 W/cm$^{2}$).

In single-array intracranial results as shown in Fig. 4, CORB markedly improves neuromodulation resolution compared with two benchmark methods. The OTAA of CORB is approximately 68.4 mm$^{2}$ in Fig. 4f, which is a $64.6\%$ improvement compared with the off-target suppression benchmark. In contrast to Fig. 3, the intracranial stimulation has higher transducer energy efficiency than the transcranial stimulation. Another advantage of intracranial neuromodulation is that it prevents high intensity in supportive tissue, such as skin and skull, which is significant in transcranial neuromodulation. Because the array is very close to brain tissue, however, it is even more difficult to eliminate all off-target excitation in the intracranial scenario with only one transducer array.

**Figure 5:**
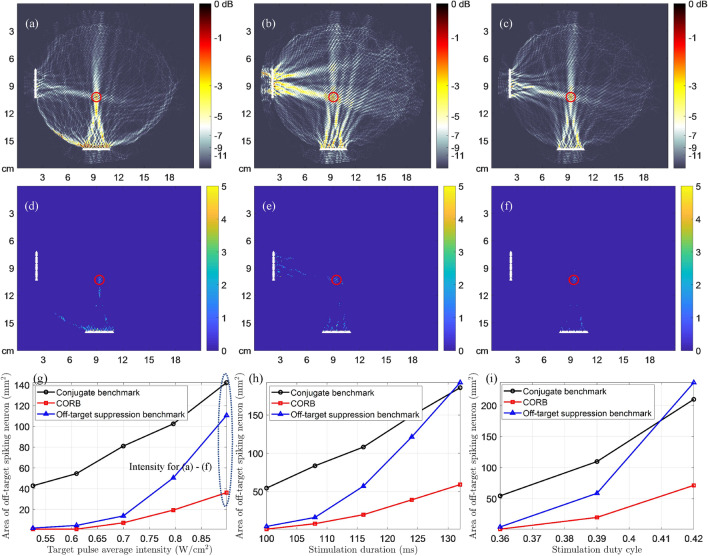
The beam patterns and neuronal spike patterns of intracranial stimulation with double transducer arrays, demonstrating the minimized OTAA of CORB compared with two benchmarks. The location of the target is the same as in Fig. 4. The distance between the target point and the left array is approximately 7.35 cm. (**a**–**c**) Approximate beam patterns during a pulse of $T = 0.36$ ms generated by the conjugate benchmark, the off-target suppression benchmark, and CORB, respectively^3^. The color maps demonstrate the spatial distribution of mean ultrasound intensity normalized by the target signal intensity in the pulse (0.90 W/cm$^{2}$)^22^. (**d**–**f**) Neuronal spike count map of conjugate benchmark, the off-target suppression benchmark, and CORB, respectively, in a pulsed-wave LIFU stimulation (target intensity: 0.90 W/cm$^{2}$; duration: 100 ms; duty cycle: $36 \%$; PRF: 1 kHz). Ultrasound pressures at all other points are scaled proportionally to the target based on the mean intensity distributions in (**a**–**c**). (**g**) The OTAA with different stimulation intensities (duration: 100 ms; duty cycle: $36 \%$; PRF: 1 kHz). (**h**) The OTAA with different stimulation durations (target intensity: 0.61 W/cm$^{2}$; duty cycle: $36 \%$; PRF: 1 kHz). (**i**) The OTAA with different duty cycles given the fixed pulse duration (target intensity: 0.61 W/cm$^{2}$; duration: 100 ms).

**Figure 6:**
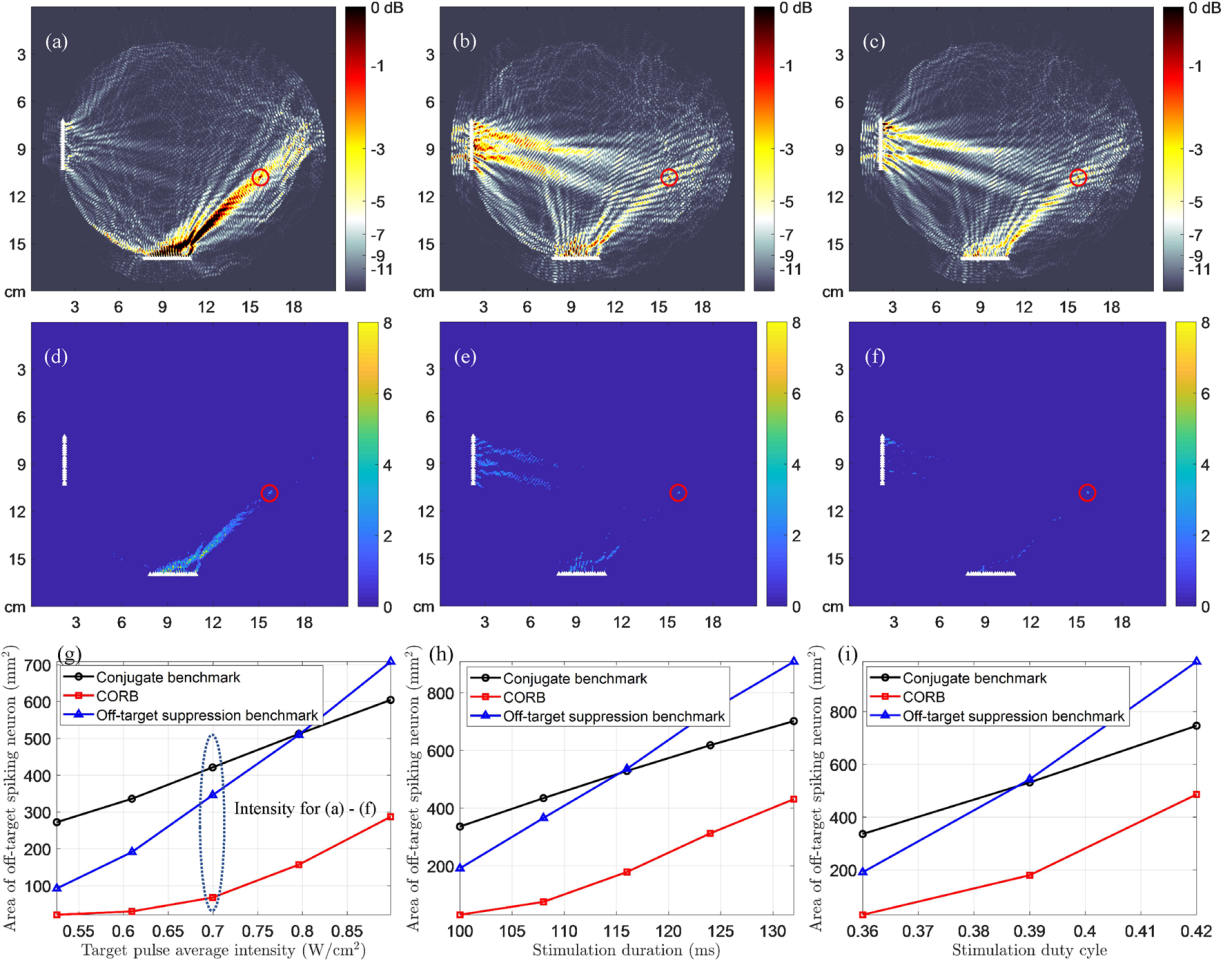
The beam patterns and neuronal spike patterns of intracranial stimulation using double transducer arrays, demonstrating the minimized OTAA of CORB compared with two benchmarks. The distance from the target point to the bottom array is approximately 8.18 cm, and the distance to the left array is approximately 13.73 cm. (**a**–**c**) Approximate beam patterns during a pulse of $T = 0.36$ ms generated by the conjugate benchmark, the off-target suppression benchmark, and CORB, respectively^3^. The color maps demonstrate the spatial distribution of mean ultrasound intensity normalized by the target signal intensity in the pulse (0.70 W/cm$^{2}$)^22^. (**d**–**(f)**) Neuronal spike count map of conjugate benchmark, the off-target suppression benchmark, and CORB, respectively, in a pulsed-wave LIFU stimulation (target intensity: 0.70 W/cm$^{2}$; duration: 100 ms; duty cycle: $36 \%$; PRF: 1 kHz). Ultrasound pressures at all other points are scaled proportionally to the target based on the mean intensity distributions in (**a**–**c**). (**g**) The OTAA with different stimulation intensities (duration: 100 ms; duty cycle: $36 \%$; PRF: 1 kHz). (**h**) The OTAA with different stimulation durations (target intensity: 0.61 W/cm$^{2}$; duty cycle: $36 \%$; PRF: 1 kHz). (**i**) The OTAA with different duty cycles given the fixed pulse duration (target intensity: 0.61 W/cm$^{2}$; duration: 100 ms).

In double-array intracranial results as shown in Fig. 5, CORB markedly reduces the OTAA to about 36.2 mm$^{2}$ in Fig. 5f without delivering high energy to supportive tissue. Compared with the off-target suppression benchmark, CORB reduces the OTAA by around $67.3\%$. Compared with the single-array setups, the double-array setup allows for much less unintended neuronal excitation for the same target point. Although more surgically invasive, the crossing beam can be a valuable option for future clinical applications. The performance of CORB with a farther target point in brain tissue is also evaluated in Fig. 6. In Fig. 6f, CORB reduces the OTAA to about 67.9 mm$^{2}$, which is an $80.4\%$ improvement over the off-target suppression benchmark. This result implies that an implanted double-array transducer system can stimulate most regions inside the skull.

### Comparison of continuous-wave and pulsed-wave stimulation

When pulsed-wave LIFU is used for stimulation in Fig. 5f, the neuronal spiking response at the target point is weaker given similar neuromodulation resolution to continuous-wave stimulations in Figs. 3f and 4f. Such a phenomenon is attributed to the different dependence of neuronal spiking responses on continuous-wave and pulsed-wave intensities. When the neuromodulation task aims to eliminate any unintended neuronal excitation, $I_{l}$ needs to be set based on the neuronal excitation intensity thresholds in Fig. 2 ($\sim 0.05$ W/cm$^{2}$ for continuous wave or $\sim 0.45$ W/cm$^{2}$ for pulsed wave). Compared with the continuous-wave stimulation, the number of neuronal spikes elicited by pulsed-wave stimulation increases slower and non-monotonically with intensity. As a result, given the same $\frac{I_{l}}{I_{h}}$ value that determines the OTAA of CORB method, the target neuron with a LIFU intensity of $I_{h}$ spikes less frequently during pulsed-wave stimulation. However, if the neuromodulation task tolerates minor unintended neuronal excitation, larger LIFU intensities (e.g., higher than 3.2 W/cm$^{2}$) can be used for pulsed-wave stimulation. Note that these results depend on the duty cycle of the pulsed wave. Simulations can show that pulsed waves with high duty cycles, e.g., $70 \%$, can be more energy efficient and generate more neuronal spikes in some scenarios^15^. This property implies that the trade-off between target neuronal excitation and unintended neuronal excitation is more difficult to achieve with pulsed-wave stimulation of low duty cycles.

### Dependence of OTAA on LIFU parameters

Subfigures (g)–(i) in Figs. 3, 4, 5, 6 show the dependency curves of the OTAA on LIFU intensity, duration, and duty cycle, respectively, with the three beam focusing methods. The OTAAs generated by different beam focusing algorithms generally increase with the LIFU intensities, durations, and duty cycles, regardless of the waveforms used and the transducer array placements. In all scenarios, CORB consistently has the lowest OTAA value with any intensity, duration, and duty cycle applied. The off-target suppression benchmark outperforms the conjugate benchmark when the intensity, duration, and duty cycle values are small. However, as these LIFU parameters increase, the off-target suppression benchmark has worse performances and can even be outperformed by the conjugate benchmark with pulsed-wave double-array intracranial stimulation.

### Lower bound evaluation of OTAA

**Figure 7:**
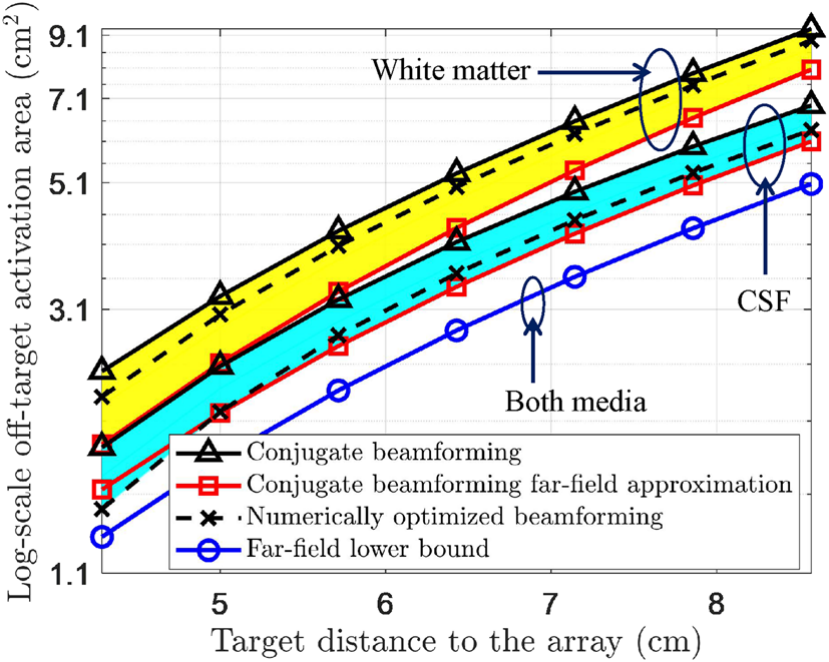
Approximated OTAA and lower bound plotted in log-scale for a single-array system with analytical approximations in homogeneous white matter/CSF. Solid black curves denote the actual $S(\varvec{w})$ of the conjugate benchmark. The optimum of formulation (5) is denoted by dashed black curves. The red curves denote the intermediate far-field approximation (S-7) of the conjugate benchmark. The blue curve denotes the lower bound $S_{LB}$.

In Fig. 7, the theoretical lower bound of OTAA, $S_{LB}$, is computationally validated for different media and target locations by comparing with the the conjugate benchmark and optimal beamforming. As is shown in the log-scale plot, an intermediate approximation to $S(\varvec{w})$ in (S-9) follows the trend of the actual $S(\varvec{w})$ using the conjugate benchmark in either CSF or white matter. The approximation error is mainly caused by the near-field beam pattern. The lower bounds in the CSF and white matter nearly overlap, as the influence of attenuation is mostly omitted in the approximation in (S-9) in the Supplementary Information. The derived lower bound is not violated in either medium, implying that the theoretical analysis is valid for evaluating the feasibility of neuromodulation resolution requirements. In real-world LIFU procedures, given the desired neuromodulation resolution, transducer parameters need to be carefully optimized to ensure the required OTAA is in the region above the blue curve. Since the derived bound assumes a homogeneous medium, the skull effect, either in the form of refraction or reflection, is not considered. Hence, this bound should be applied in scenarios in which the skull effect is relatively weak (e.g., in intracranial stimulations as in Fig. 4).

## Discussion and conclusion

The current study introduces novel methodology involving the use of the OTAA to evaluate unintended neuronal excitation during LIFU neuromodulation and proposes the CORB method for minimizing unintended neuronal excitatory effects while ensuring the excitation of target neurons. The lower bound of OTAA is theoretically derived and validated computationally. Simulations within a 2-D human head model are conducted to numerically evaluate the OTAA of three beam focusing methods with three different transducer setups. Numerical results show that the OTAA increases with stimulation intensity, duration, and duty cycle. Also, the proposed CORB method can significantly reduce areas with unintended neuronal spiking responses compared with benchmark methods using all three array setups.

Simulation results show that LIFU is a promising technique for high-resolution neuromodulation with reduced invasiveness in clinical applications. Although the resolution metric is difficult to unify, a simple comparison between this work and previous results reported in the literature implies that LIFU with CORB may achieve higher neuromodulation resolution than many other neuromodulation techniques. Methods based on transcranial electrical currents^27^ typically include modulation areas from greater than 20 mm$^{2}$ to the cm$^{2}$ scale^28,29^. The resolution of transcranial magnetic stimulation in each dimension is also usually on a cm scale^30^. Traditional ultrasound neuromodulation methods, either with single-element or phased array transducers, have focusing mechanisms similar to the conjugate benchmark, which is significantly outperformed by the proposed CORB method. The achievable FWHM resolution of traditional LIFU neuromodulation in human applications is usually several millimeters laterally and can achieve cm scale along the transducer axis, given the low frequency range (250–500 kHz)^3,4,16^. The estimated equivalent OTAA, given the higher stimulation intensity used^3^ and the simulated neuronal response patterns in Fig. 2, can be even larger. In contrast, the CORB method achieves only 3.1 mm$^{2}$ modulation area using non-invasive stimulation, which is much more focused than other methods. The reason for the observed improvement is that the CORB method optimizes the overall ultrasound intensity distribution in the entire brain, whereas traditional focusing techniques only consider the target point. Other neuromodulation techniques, including deep brain stimulation and optical methods, can achieve cell-level stimulation but are highly invasive. Hence, LIFU with CORB could serve as a less-invasive tool for future clinical treatment.

This study has also sought to establish stimulation parameters and protocols for clinical applications involving neuromodulation resolution requirements. By simulating the neuronal spike pattern with the SONIC model (Fig. 2), the study presents intensity threshold values (e.g., $\sim 0.05$ W/cm$^{2}$ for continuous wave and $\sim 0.45$ W/cm$^{2}$ for pulsed wave) that can optimally suppress potential side effects caused by unintended neuronal excitation in clinical trials. In addition, in the aspect of neuronal susceptibility to stimulation, the results of this work suggest that continuous-wave stimulation has an advantage over pulsed-wave stimulation of low duty cycles with respect to reducing unintended neuronal excitation when standing wave effects can be approximately modeled. The advantages of different transducer setups have been revealed in simulation results. The transcranial single-array setup can successfully stimulate the target with minimal unintended neuronal excitation in a non-invasive manner with the CORB method, but has low transducer energy efficiency and potential side effects on supportive tissue. The intracranial single-array setup can deliver the most energy to the target but has relatively limited neuromodulation resolution. The intracranial double-array setup minimizes the impact to both off-target brain tissue and supportive tissue at the expense of increased invasiveness. Currently most research use single-element transducers for transcranial stimulation. Although intracranial stimulation, especially the multi-array setup, is the minority so far, researchers have been continuously exploring methods to avoid skull effects in ultrasound procedures. E.g., a work^31^ has created a cranial window so that the transducer can be placed directly on dura. Another work^32^ investigates ultrasound transducer miniaturization for implantation, and has proposed to consider implanting multiple transducers into a rat. Literature^33^ has already shown the high resolution of multi-array transducer setups as well. Therefore, despite few current research using intracranial multi-array transducer setups, this direction could be explored in the future, and the proposed CORB method should be able to support this setup.

The high-resolution neuronal excitation effect demonstrated in this study could potentially be used to treat neuropsychiatric disorders. For example, a previously reported therapy for Parkinson’s disease stimulates the internal globus pallidus with electrical currents, with the probable mechanism being suppression of the thalamus activity via excitation of the internal globus pallidus neurons^34,35^. Given the similar excitatory effect of LIFU, high-resolution focus of ultrasound energy to the internal globus pallidus using CORB may serve as a non-invasive treatment for Parkinson’s disease after experimental validations.

The fundamental mechanism of ultrasonic neuromodulation is still not completely clear. Although the definition and evaluation of OTAA in this work is based on SONIC model, the results, e.g., comparison of OTAA between different methods, will not be qualitatively different when other ultrasonic neuromodulation mechanisms are involved. The reason is that CORB method only relies on two intensity thresholds, $I_{h}$ and $I_{l}$, for transducer beam optimization based on neuronal responses. Many experimental results have shown the existence of ultrasound pressure/intensity/radiation force thresholds for neuronal activation, regardless of the mechanism proposed to explain such phenomena, and the threshold metrics are mostly simple functions of the ultrasound pressure. Therefore, CORB can be easily generalized to support other mechanisms with neuronal excitation thresholds provided.

The proposed computational model of unintended neuronal excitation does not address other important considerations in evaluating neuromodulatory outcomes, including other types of neuromodulation effects, differential effects across brain regions, and the impact of brain anesthesia state on proper neuromodulation resolution metrics. Recent reports of the complexity of neural responses to ultrasound stimulation can benefit the generalization of the model through integration of corresponding excitation/inhibition mechanisms, such as the neuron-type-selectivity of responses to ultrasound PRF^36^. The SONIC model supports the spike pattern simulation of multiple types of neurons including subthalamic nucleus neurons, which allows the OTAA model to account for brain region specificity^11^. In addition, neuronal activation elicited by ultrasound stimulation can be subsequently transmitted to other functionally connected brain regions^37^. Thus, a comprehensive understanding of the spike-level response of brain circuits to ultrasound stimulation could guide the design of beam focusing techniques.

To better approximate the *in vivo* setup and further validate the effectiveness of the proposed method, future work could extend the current framework to 3-D scenarios and phantom experimental setups. The skull is also modeled as homogeneous medium based on one-layer assumption in this work, leaving heterogeneity to be considered in the next step^38^. In addition, the acoustic parameter selection and computational accuracy can influence the effectiveness of the designed beam pattern^39^. Thus, a sensitivity analysis of neuromodulation resolution based on the acoustic parameters of brain tissue will be the focus of future studies.

### Supplementary Information

Supplementary Information.

## Data Availability

The brain model dataset used in the current study is available in BrainWeb: Simulated Brain Database, https://brainweb.bic.mni.mcgill.ca/. This work is based on a conference presentation^10^ in 2020.
