# Supplementary material for: Computational modeling and minimization of unintended neuronal excitation in a LIFU stimulation
Source: Sci Rep. 2023 Aug 17;13:13403. doi: 10.1038/s41598-023-40522-w (PMC10435497; doi:10.1038/s41598-023-40522-w)
Supplement: Supplementary file 1 — Supplementary Information. [file 41598_2023_40522_MOESM1_ESM.pdf]

Supplementary Information

Computational Modeling and Minimization of Unintended Neuronal Excitation in a LIFU Stimulation

Boqiang Fan[1,*], Wayne Goodman[1,2], Raymond Y. Cho[2], Sameer A. Sheth[1,3], Richard R. Bouchard[4], and Behnaam Aazhang[1]

[1]Department of Electrical and Computer Engineering, Rice University, Houston, TX, 77005, USA
[2]Department of Psychiatry and Behavioral Science, Baylor College of Medicine, Houston, TX, 77030, USA
[3]Department of Neurosurgery, Baylor College of Medicine, Houston, TX, 77030, USA
[4]Department of Imaging Physics, University of Texas MD Anderson Cancer Center, Houston, TX, 77030, USA
[*]Boqiang.Fan@rice.edu

ABSTRACT

The supplementary information contains detailed information regarding the setup of computational evaluations and mathematical derivations of CORB method and the lower bound of OTAA. Equations and figures from the article are referred in the format of, e.g., equation (M-1) and Fig. M-1. Equations and figures in this supplementary information is referred to by numbers. The references in the article and the supplementary information are separately numbered.

Computational Evaluation Setup

Human Head Model

A 2-D cross-section of a human head model as shown in Fig. 1 is used for the evaluation of unintended neuronal excitation effects during LIFU neuromodulation. This human head model, which consists of skin, muscle, connective tissue, fat, skull, cerebrospinal fluid (CSF), and grey and white matter, is generated using magnetic resonance imaging data from a previous study[1]. According to literature[2], this model classify CSF volumes outside of brain tissue region also as skull or skin, which leads to skin volumes inside skull, as is shown in Fig. 1. Although real-world LIFU neuromodulation is performed in a 3-D human head, the current analysis is conducted in a 2-D environment to maintain manageable computational loads. Further, the results of this study can be extended to 3-D environments with additional computational complexity. The brain tissue region for neuromodulation includes the CSF and grey and white matter, which contains many neurons susceptible to LIFU stimulation. The media in other supportive tissues, including fat, skin, muscle, connective tissue, and the skull, are assumed to not be significantly impacted by ultrasonic waves during stimulation, regardless of the LIFU parameters. To evaluate ultrasound propagation, the environment space is discretized and interpolated into grid points G[3]. The grid size is ~ 0.143 mm and the CFL number is set to 0.1, which satisfies the stability condition and is sufficiently small to reduce numerical errors in ultrasound propagation computations.

k-Wave Ultrasound Propagation Model

The validated k-Wave toolbox[4] is used to model ultrasound propagation in a human head, which numerically solves the modified Westervelt equation[5] using a pseudospectral method. The numerical solution depicts the acoustic pressure $p(G,t)$ over time t at any grid point G in the space given source signals at each array element, which can be used for both beam pattern design and evaluation. When the stimulation pressure is limited, the nonlinear effect of ultrasound propagation is insignificant[5,6]. The elasticity of the skull is not modeled to maintain manageable computational loads, and the incident angle of the transcranial array setup is small[7]. The comparative beam focusing schemes investigated in this study do not rely on the elasticity assumption and can be generalized in a straightforward manner.

The brain tissue acoustic parameters for k-Wave simulation input include the ambient medium density $\rho(G)$, the sound speed $c(G)$ at the desired frequency, and the attenuation coefficient $\alpha(G)$ that uses the frequency-dependent power law modeling. A summary of desired acoustic parameters for different media, converted from published values[6,8–18], is listed in Table 1. The unit for f is MHz, and in this work the frequency is set to $f = 0.5$ MHz[19]. The conversion is based on the property of the wave equation described in a previous work[20]. A more recent work[21] has suggested a different way of converting the attenuation coefficients. According to the suggested conversion[21], the older conversion[20] has some inaccuracy, which has been evaluated in this work. Based on the model[21], at $f = 0.5$ MHz, the gap between exhibited attenuation coefficients in k-wave simulations and

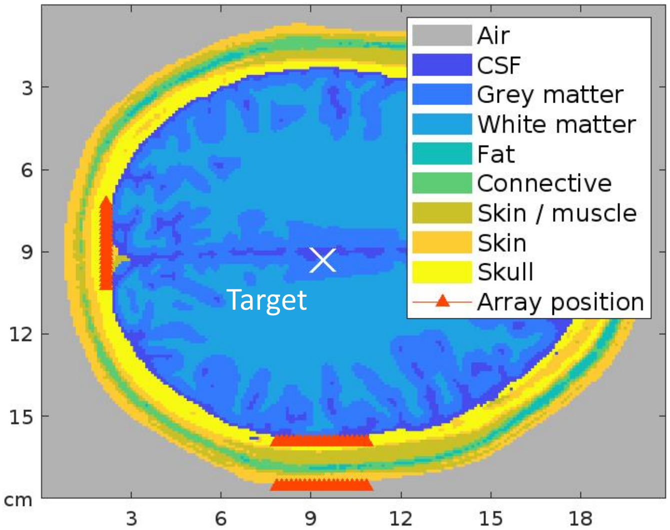

Figure 1. A 2-D human head model with a target point denoted by the white cross[1]. Potential positions for phased arrays are denoted by red triangles, and three phased array setups are evaluated in this work: single array for transcranial stimulation, single array at the bottom for intracranial stimulation, and double array for intracranial stimulation.

Table 1. Acoustic parameters of media used in k-Wave beam pattern simulations. Due to the limitation of the k-Wave tool box, a homogeneous power law coefficient, 1.08 (for gray / white matter), is assumed for all media. The attenuation and sound speed values in the table are desired in k-Wave simulations at frequency $f = 0.5$ MHz given power-law frequency dependency[20].

Medium at point G	Density $\rho(G)$ (kg/m^3)	Sound speed $c(G)$ (m/s)	Attenuation $\alpha(G)$ (dB/cm)
Air	1.2041	343.21	$0.8879f^{1.08}$
CSF	999.5	1483	$0.0037f^{1.08}$
Grey matter	1045	1539	$0.7500f^{1.08}$
White matter	1041	1543	$0.9700f^{1.08}$
Fat	950	1450	$0.6342f^{1.08}$
Skin / muscle / connective tissue	1150	1730	$1.8087f^{1.08}$
Skull	1912	2850	$14.8735f^{1.08}$

the desired values in Table 1 should be less than 5% in brain tissue regions (CSF, white matter and grey matter). Therefore, such inaccuracy has very limited impact on intracranial simulations considered in this work. The gap of attenuation coefficients in the skull, on the other hand, can be much larger (~ 3 dB / cm). As the CORB method does not rely on any assumed acoustic property of the human head tissues, the performance of CORB will not be qualitatively altered by the conversion methods used and the main goals of this work will not be invalidated.

The total amplitude and phase changes from an element m to a point G, $h_m(G,t)$, can be extracted from k-Wave simulation result $p_m(G,t)$ using the Hilbert transform with the mirror-extending method to mitigate the end effects at the beginning and end of the signal[22]. As illustrated in an example scenario shown in Fig. 2, $h_m(G,t)$ oscillates before $t_c = 0.7$ ms due to the superposition of incident and reflected waves, and becomes stable after t_c. Such a phenomenon also occurs for transcranial stimulation, because the amplitude of residual reflective waves, which are continually attenuated by the absorptive media, becomes insignificant. Given the observation, a k-Wave simulation duration of $T = 2$ ms is long enough to approximate a continuous-wave beam pattern. In addition, when k-Wave simulation is used to generate $h_m(G,t)$ for beam focusing optimization, the simulation duration is set to at most $t_c = 0.7$ ms, since the stable $h_m(G,t)$ around t_c can be used to approximate the long-term average propagation.

Phased Array Transducer Model

Single-element focused transducers have been widely used in ultrasound neuromodulation experiments, but cannot support flexible focal points and high neuromodulation resolution given limited targeting control[23]. Phased array transducers can provide high targeting precision and flexibility[24], and can be even more appealing for continuous treatment of neuropsychiatric disorders if portability is also supported[25]. The current study assumes wearable phased array transducers are used for neuromodulation, which can be transcranially or intracranially planted and allow beam steering for flexibly sonicating different target regions

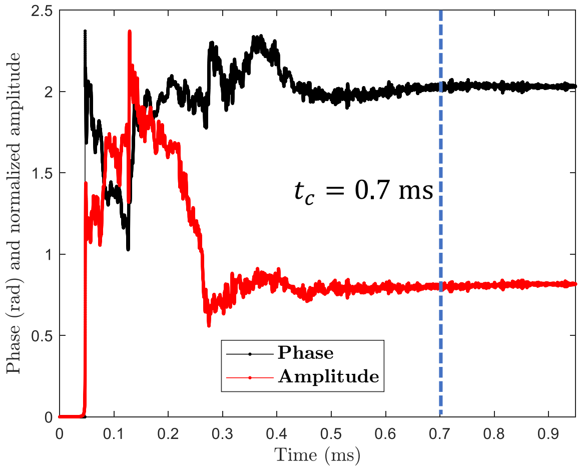

Figure 2. Simulated changes in the amplitude and phase of $h_m(G,t)$ over time for an intracranial transducer element m and a deep brain tissue region G. The delay at the beginning denotes the period before the wave from m propagates to G. The wave at G becomes stable after approximately $t_c = 0.7$ ms.

with high focusing resolution[26,27].

The three transducer setups considered in this work are shown in Fig. 1. Each phased array consists of 20 array elements and is about 2.99 cm wide. Although the transcranial transducer setup is widely used in most experimental research due to its noninvasive nature, one challenge for transcranial ultrasound stimulation protocols is phase aberration through the skull, which can substantially diminish transmit-focusing[7]. At the expense of invasiveness, the intracranial setup effectively avoids the confounding effects of the skull and may be more suitable for continuous treatment for neuropsychiatric disorders by mitigating potential social stigma due to the presence of an unsightly external array[26]. The double-array setup uses two uniform linear arrays with the same number of array elements and strives to further improve focusing resolution by providing crossing ultrasound beams for increased energy-focusing[28]. One important application of a double-array system is to better support multi-focus neuromodulation in situations in which different brain regions are required to be stimulated simultaneously to activate a brain circuit[29]. A large aperture is usually required in this case[29], but cannot be implanted intracranially for real-time stimulation. Conversely, the intracranial double-array system evaluated in this study can potentially support real-time multi-focus stimulation using adapted beamforming algorithms.

Beam Focusing Methods

Traditional beam focusing schemes were not specifically designed for minimizing unintended neuronal excitation in LIFU neuromodulation. For example, the time-reversal method[30] maximizes signal intensity at the target point without considering other regions by compensating for propagation attenuation and phase shifts from phased array elements. Some algorithms are designed to control the lobes of the beam, including an approach that aims to suppress the side lobes of the beam while maintaining a relatively high pressure at the focal point[31]. Methods to approximately generate desired beam patterns on the focal plane have also been proposed[32,33]. Another approach maximizes signal intensity at the target point while maintaining an upper-bound mask for signal intensity in all other regions[34]. To evaluate the performance of the proposed CORB method, this work uses the two following benchmark beam focusing algorithms: the conjugate beamforming benchmark and the off-target suppression benchmark.

Conjugate Benchmark

The conjugate benchmark beamforming vector is $w_{conj} = \mathbb{P}(v(G^*))$, where $v(G^*)$ denotes the eigenvector of $Q(G^*)$ that corresponds to the largest eigenvalue.

Off-Target Suppression Benchmark

The concept has been widely used in applications including cancer treatment and transcranial electric stimulation[34,35]. For the ultrasound neuromodulation setup, the benchmark beamforming vector, defined as w_{OTS}, is the normalized solution of the

following optimization problem,

$$\max_{w\in\mathbb{C}^M} \quad I(G^*), \text{ s.t. } I(G) \leq I_{th}, \; \forall\, G \neq G^*. \tag{1}$$

Here I_{th} is an upper limit of ultrasound intensity for off-target regions, and without losing generality, is set to $I_{th} = \max_G [w_{conj}]^H Q(G) w_{conj}$. The MATLAB solver is used to solve for w_{OTS}, with the initialization beamforming vector being w_{conj}.

CORB

Some mathematical details on the derivation of CORB method is introduced in the Supplementary Information. As is mentioned in the article, the equivalence of minimizing (M-3) and (M-4) is proved in Appendix 1. In addition, the value of μ in (M-5) determines if the global optimum of the approximated formulation (M-5) is the same as the optimal beam pattern that minimizes $S(w)$. The threshold for the logistic function steepness μ that sufficiently maintains optimality remains unclear. Thus, a guideline lower bound $\mu \geq \mu_{min}$ is derived in (18). For beam pattern computations this work uses a larger value $\mu = 10\mu_{min}$.

Parameter values of CORB method used in this work are listed as follows. Across multiple trials, a step-size of $\gamma = 10^{-6}$ Pa^2 and iteration-ending threshold of $\beta = 10^{-4}$ Pa achieves the best performance in CORB optimization, given a normalized $P = 1$ Pa^2. In Fig. M-3 to M-6, the neuronal excitation threshold ratio is set to $\frac{I_l}{I_h} = 0.65$[36]. CORB uses $h(G,t)$ of each brain tissue region G as input, which is acquired by post-processing ultrasound propagation from each transducer element to brain tissue region in k-Wave simulation[37] in the human head model.

The CORB method can be further extended to jointly optimize transducer beam, power and stimulation duration. Based on the formulation (M-4), the generalized formulation including transducer power and stimulation duration is as follows:

$$\min_{w\in\mathbb{C}^M, P, T} \quad \sum_{G\neq G^*} \chi\left([w]^H Q^*(G,T) w\right), \text{ s.t. } ||w||_2^2 = P,\; Q^*(G,T) = Q(G,T) - \frac{I_l(T)}{I_h(T)} Q(G^*,T). \tag{2}$$

Here the propagation matrix $Q(G,T)$ is a function of stimulation duration T, especially for a pulsed wave stimulation. The neuronal excitation thresholds, as demonstrated by SONIC model, also change with the stimulation duration. The on-target stimulation efficiency can also be integrated as a constraint of $[w]^H Q(G^*,T) w/P$. On the other hand, the computational complexity for solving this generalized formulation will be higher.

Theoretical Lower Bound of OTAA

The detailed derivation of OTAA lower bound is presented as following. Although a real human head model is preferred in OTAA analysis, heterogeneity makes it difficult to analytically model refraction and reflection. Hence, the simplified analysis considers the proposed beam focusing framework in ideal homogeneous brain tissue medium such as CSF, grey matter or white matter. As shown in Fig. 3, the homogeneous medium is assumed to have an attenuation coefficient α and wavelength λ for ultrasonic signals with frequency f. The coordinate of each array element m is $(d_m, 0)$, where $d_m = \frac{\lambda}{4}(2m - M - 1)$. The distance from each point G to the center of the array is r, and the angle from the y-axis is ψ. Polar coordinates (r,ψ) can represent any point G in the space, and (r^*,ψ^*) represents the target point G^*.

In a simplified homogeneous environment, for each arbitrary point (x,y), the distance between the element m and (x,y) is $r_m = \sqrt{r^2 - 2rd_m \sin\psi + d_m^2}$. The analytic signal of the ultrasound pressure wave can be modeled in homogeneous medium as[38]

$$\tilde{p}(r,\psi) = Ae^{j2\pi ft} \sum_{m=1}^{M} [w_m]^H r_m^{-\frac{1}{2}} e^{-(\alpha + j\frac{2\pi}{\lambda}) r_m}, \tag{3}$$

where $A = 1$ is assumed without loss of generality. A cylindrical wave model is applied because a 2-D scenario is considered. According to (M-4), the off-target excitation region is equivalent to the region with (r,ψ) satisfying $|\tilde{p}(r,\psi)|^2 \geq \frac{I_l}{I_h} |\tilde{p}(r^*,\psi^*)|^2$. To derive the condition of (r,ψ) for off-target excitation, the analysis is further simplified by considering a far-field approximation based on one simple criterion from[39] (i.e., the distance to the array $r \gg |d_M|$). Such an approximation can be applied to scenarios in which the array must be very small due to biological space constraints. A Taylor series expansion to r_m regarding $\frac{d_m}{r} \to 0$ is $r_m = r - d_m \sin\psi + \cdots$, used to approximate r_m and pursue a far-field representation. As a result, without integrating the medium density and sound speed, the square of the pressure, which is proportional to intensity, is approximately equal to

$$|\tilde{p}(r,\psi)|^2 \approx r^{-1} e^{-2\alpha r} \left| \sum_{m=1}^{M} [w_m]^H e^{(\alpha + j\frac{2\pi}{\lambda}) d_m \sin\psi} \right|^2. \tag{4}$$

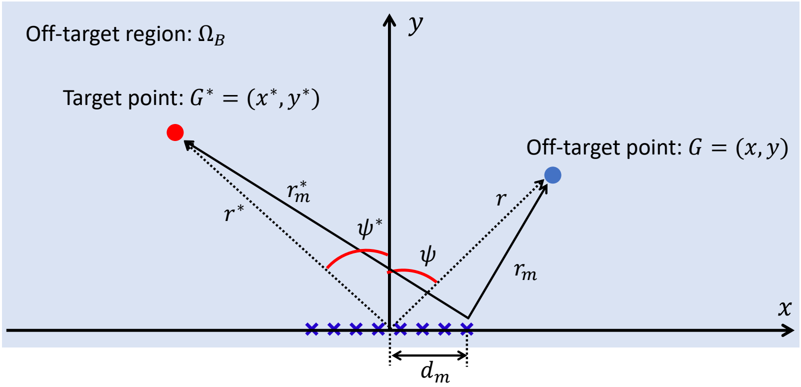

Figure 3. The geometry of single-array beamforming in simplified homogeneous medium used for estimating OTAA lower bound. The continuous region subject to the stimulation effect is defined as the entire region with coordinates $y \geq 0$. Only one uniform linear array with half-wavelength element spacing is located on the x-axis, centered at $(0,0)$.

Given (4), any activated point (r,ψ) satisfies $|\tilde{p}(r,\psi)|^2 \geq \frac{I_l}{I_h}|\tilde{p}(r^*,\psi^*)|^2$, which is equivalent to

$$re^{2\alpha r} \leq \frac{I_h}{I_l|\tilde{p}(r^*,\psi^*)|^2}\left|\sum_{m=1}^{M}[w_m]^H e^{(\alpha+j\frac{2\pi}{\lambda})d_m \sin\psi}\right|^2. \tag{5}$$

The left term in (5) is an increasing function of r. Therefore, given any direction ψ, there exists a maximal distance $R(\psi)$ such that all closer points with $r \leq R(\psi)$ will be activated. The off-target excitation region is the entire area inside the contour $r = R(\psi)$. Using the main branch of the Lambert W function, $W_0(\cdot)$, which satisfies $W_0(z)e^{W_0(z)} = z$[40], a closed-form expression of $R(\psi)$ is derived as

$$R(\psi) = \frac{1}{2\alpha}W_0\left(\frac{2\alpha I_h}{I_l|\tilde{p}(r^*,\psi^*)|^2}\left|\sum_{m=1}^{M}[w_m]^H e^{(\alpha+j\frac{2\pi}{\lambda})d_m \sin\psi}\right|^2\right). \tag{6}$$

The area $S(w)$ can be approximated by

$$S(w) \approx \frac{1}{2}\int_{-\frac{\pi}{2}}^{+\frac{\pi}{2}} R^2(\psi)d\psi. \tag{7}$$

A lower bound of $S(w)$ is further derived based on the approximation in (7). By further assuming weak attenuation where α in (5) is very small (i.e., $e^{-2\alpha r} \approx 1$), the simplified excitation condition for r can be acquired as

$$r \leq R(\psi) \approx \frac{r^* I_h \left|\sum_{m=1}^{M}[w_m]^H e^{(\alpha+j\frac{2\pi}{\lambda})d_m \sin\psi}\right|^2}{I_l\left|\sum_{m=1}^{M}[w_m]^H e^{(\alpha+j\frac{2\pi}{\lambda})d_m \sin\psi^*}\right|^2}. \tag{8}$$

Combining (7), (4) and (8), $S(w)$ is approximately equal to

$$S(w) \approx \frac{(r^*)^2 I_h^2 \int_{-\pi}^{+\pi}\left|\sum_{m=1}^{M}[w_m]^H e^{(\alpha+j\frac{2\pi}{\lambda})d_m \sin\psi}\right|^4 d\psi}{4I_l^2\left|\sum_{m=1}^{M}[w_m]^H e^{(\alpha+j\frac{2\pi}{\lambda})d_m \sin\psi^*}\right|^4}. \tag{9}$$

The integral here can be extended to $(-\pi,\pi)$ as the beam pattern is symmetric about the x-axis. To solve for an OTAA lower bound, (9) is minimized with relaxation and conversion to a problem similar to array gain optimization[41]. The terms in (9) are rearranged for summation by defining k_p as

$$k_p = \begin{cases} \sum_{m=1}^{p} w_m w_{p-m+1}, & p = 1,\cdots,M \\ \sum_{m=p-M+1}^{M} w_m w_{p-m+1}, & p = M+1,\cdots,2M-1 \end{cases} \tag{10}$$

Then by defining the vector $K = [k_1, \cdots, k_{2M-1}]^H$, the area function in (9) can be rewritten as

$$S(K) \approx \frac{(r^*)^2 I_h^2 K^H Q^J K}{4 I_l^2 K^H H(\psi^*)[H(\psi^*)]^H K}. \tag{11}$$

Here, $H(\psi^*) = [H_1(\psi^*), \cdots, H_{2M-1}(\psi^*)]^H$. For any p, $H_p(\psi^*) = e^{(\alpha + j\frac{2\pi}{\lambda})\frac{(p-M)\lambda}{2}\sin\psi^*}$. $H(\psi)$ is defined similarly, and Q^J is defined as

$$Q^J = \int_{-\pi}^{+\pi} H(\psi)[H(\psi)]^H d\psi. \tag{12}$$

The square matrix Q^J has dimensions of $2M-1$, with element Q^J_{pq} on the pth row and qth column calculated with the zero-order Bessel function[42],

$$Q^J_{pq} = \int_{-\pi}^{+\pi} [H_p(\psi)]^H H_q(\psi) d\psi = \int_{-\pi}^{+\pi} e^{\sin\psi(\frac{\alpha\lambda(p+q-2M)}{2} - j\pi(q-p))} d\psi = 2\pi J_0(-\pi(q-p) - j\frac{\alpha\lambda(p+q-2M)}{2}). \tag{13}$$

It can be shown that $S(K) > 0$ for any non-zero K. The denominator of (11) is a scaled norm of $K^H H(\psi^*)$, which is always non-negative. The numerator is always positive according to the following lemma, which matches the intuition, as the numerator has a similar form to the sum power over all directions.

Lemma 0.1. *The matrix Q^J is positive definite.*

Proof. Obviously $K^H H(\psi)[H(\psi)]^H K \geq 0$ for $\forall\ K \in \mathbb{C}^{2M-1}$ and $\forall\ \psi$. Therefore, the integral over ψ is $K^H Q^J K \geq 0$. For any ψ, the non-zero variable $b = e^{(\alpha + j\frac{2\pi}{\lambda})\frac{\lambda}{2}\sin\psi}$ is defined, and $b(\psi) = [b^0, \cdots, b^{2M-2}]^H$, hence $H(\psi) = [b^{1-M}]^H b(\psi)$. As the set of vectors $\{b(\psi)\}$ has a polynomial form and can formulate a full-rank Vandermonde matrix, $\{b(\psi)\}$ can serve as a group of basis vectors for the whole vector space $\mathbb{C}^{2M-1}$[43]. Therefore, for any non-zero vector K, there exists ψ such that $K^H b(\psi) \neq 0$ and $K^H H(\psi)[H(\psi)]^H K > 0$. Based on continuity, it can be concluded that $K^H Q^J K > 0$ for any non-zero vector K, which implies that Q^J is positive definite. □

With $S(K) > 0$, minimizing the OTAA is equivalent to maximizing the reciprocal of $S(K)$. The minimum of (11) can be achieved when $K \propto (Q^J)^{-1} H(\psi^*)$, and the minimum value of the OTAA is[41]

$$S_{LB} = \frac{(r^*)^2 I_h^2}{4 I_l^2 [H(\psi^*)]^H (Q^J)^{-1} H(\psi^*)}. \tag{14}$$

Due to the dependence of K on w, usually the optimal condition can not be satisfied. Therefore, S_{LB} can serve as a lower bound of the OTAA in medium with a very small attenuation coefficient α. The bound S_{LB} only depends on the location of the target point and transducer parameters, such as size and operating frequency, allowing a direct evaluation of neuromodulation resolution feasibility.

For a medium with a large α, (14) is generally no longer a strict bound. However, in the scenario with $\frac{I_l}{I_h} \to 1$, for far-field beam patterns with minimal $S(w)$, intuitively all points farther than the target from the array will not be activated (i.e., $R(\psi) \leq r^*$ for $\forall\ \psi$). Therefore, $e^{-2\alpha R(\psi)} \geq e^{-2\alpha r^*}$ for any α, which implies the approximation in (8) is usually smaller than the value of (6). Thus, the approximated $S(w)$ in (9) provides a lower bound for the area in (7), allowing the final lower bound in (14) to be valid even if the attenuation coefficient α is large. On the other hand, if α is large and $\frac{I_l}{I_h}$ is small, simulations reveal that (14) no longer serves as a valid lower bound.

In Fig. M-7, a single array with $M = 8$ elements is used to model small, implanted arrays given biological space constraints, with the same distance between adjacent elements as in previous results. The target point is located on the y-axis (i.e., $\psi^* = 0$). The excitation threshold ratio is $\frac{I_l}{I_h} = 0.99$. The plotting uses an online tool[44].

Additional Results

When the common parameter setup (target intensity: 0.61 W/cm^2; duration: 100 ms; duty cycle: 36%; PRF: 1 kHz) covered by Fig. M-5 (g)-(i) is used, the generated neuronal spike count maps are shown in Fig. 4. Both CORB method and the off-target suppression benchmark have very small OTAA. CORB method can still outperform both benchmark methods.

When the common parameter setup (target intensity: 0.61 W/cm^2; duration: 100 ms; duty cycle: 36%; PRF: 1 kHz) covered by Fig. M-6 (g)-(i) is used, the generated neuronal spike count maps are shown in Fig. 5. CORB method can still outperform both benchmark methods.

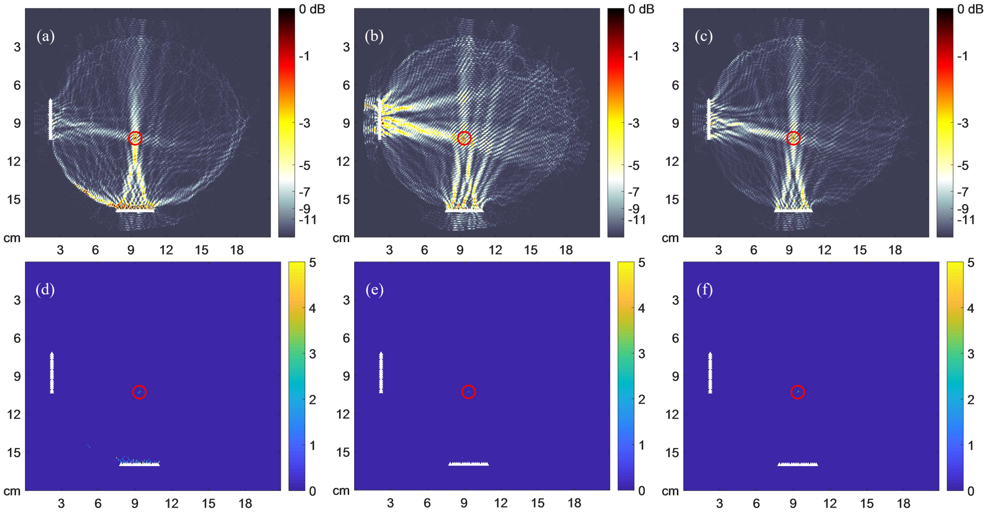

Figure 4. The beam patterns and neuronal spike patterns of intracranial stimulation with double transducer arrays, demonstrating the minimized OTAA of CORB compared with two benchmarks. (a)-(c): Approximate beam patterns during a pulse of $T = 0.36$ ms generated by the conjugate benchmark, the off-target suppression benchmark, and CORB, respectively. The color maps demonstrate the spatial distribution of mean ultrasound intensity normalized by the target signal intensity in the pulse (0.61 W/cm^2). (d)-(f): Neuronal spike count map of conjugate benchmark, the off-target suppression benchmark, and CORB, respectively, in a pulsed-wave LIFU stimulation (target intensity: 0.61 W/cm^2; duration: 100 ms; duty cycle: 36%; PRF: 1 kHz). Ultrasound pressures at all other points are scaled proportionally to the target based on the mean intensity distributions in (a)-(c).

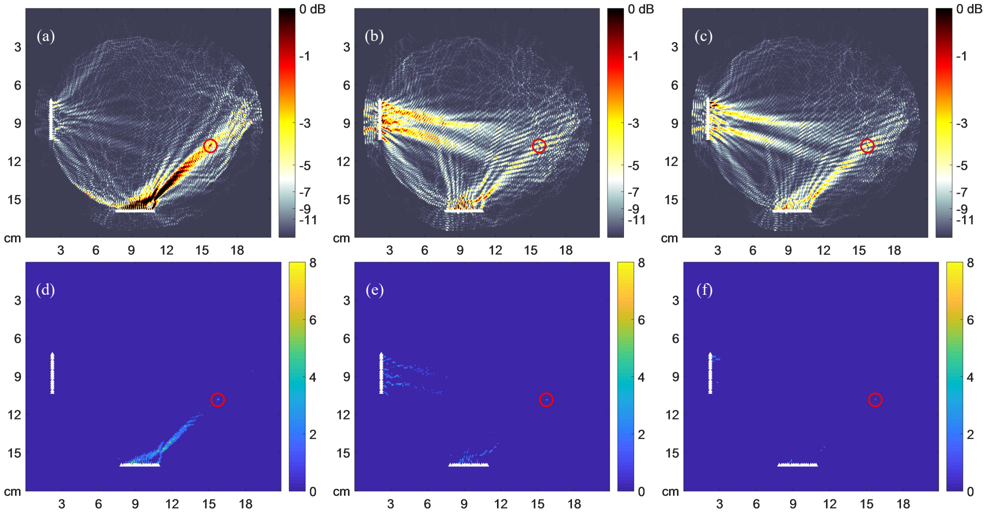

Figure 5. The beam patterns and neuronal spike patterns of intracranial stimulation using double transducer arrays, demonstrating the minimized OTAA of CORB compared with two benchmarks. (a)-(c): Approximate beam patterns during a pulse of $T = 0.36$ ms generated by the conjugate benchmark, the off-target suppression benchmark, and CORB, respectively. The color maps demonstrate the spatial distribution of mean ultrasound intensity normalized by the target signal intensity in the pulse (0.61 W/cm^2). (d)-(f): Neuronal spike count map of conjugate benchmark, the off-target suppression benchmark, and CORB, respectively, in a pulsed-wave LIFU stimulation (target intensity: 0.61 W/cm^2; duration: 100 ms; duty cycle: 36%; PRF: 1 kHz). Ultrasound pressures at all other points are scaled proportionally to the target based on the mean intensity distributions in (a)-(c).

To clearly show the comparison between continuous and pulsed waves as well as between single-array and double-array setups, the double-array beam patterns are used together with continuous-wave stimulation to simulate the neuronal spike count maps. When a continuous wave (target intensity: 0.078 W/cm^2; duration: 100 ms) are transmitted by the same beam in Fig. M-5 and Fig. M-6, the generated neuronal spike count maps are shown in Fig. 6 and Fig. 7. Compared to Fig. M-4, OTAAs in Fig. 6 are significantly reduced with the double-array setup, demonstrating the high stimulation resolution of double-array

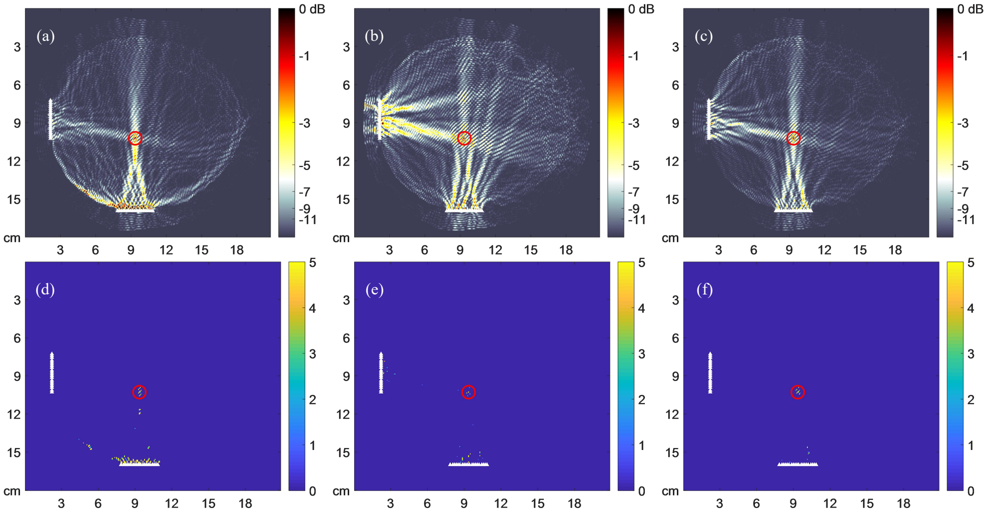

Figure 6. The beam patterns and neuronal spike patterns of intracranial stimulation with double transducer arrays, demonstrating the minimized OTAA of CORB compared with two benchmarks. (a)-(c): Approximate beam patterns for continuous-wave stimulation generated by the conjugate benchmark, the off-target suppression benchmark, and CORB, respectively. The color maps demonstrate the spatial distribution of mean ultrasound intensity normalized by the target signal intensity (0.078 W/cm^2). (d)-(f): Neuronal spike count map of conjugate benchmark, the off-target suppression benchmark, and CORB, respectively, in a continuous-wave LIFU stimulation (target intensity: 0.078 W/cm^2; duration: 100 ms). Ultrasound pressures at all other points are scaled proportionally to the target based on the mean intensity distributions in (a)-(c).

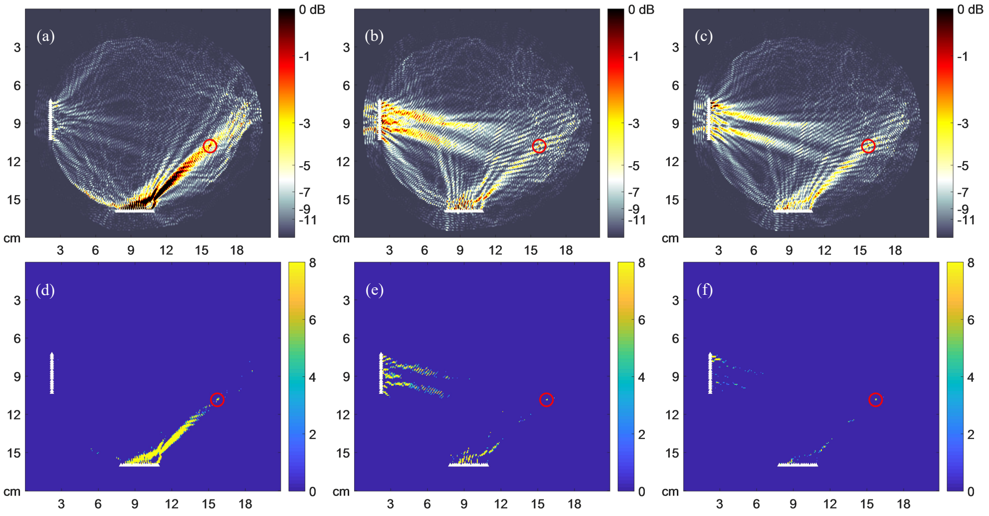

Figure 7. The beam patterns and neuronal spike patterns of intracranial stimulation using double transducer arrays, demonstrating the minimized OTAA of CORB compared with two benchmarks. (a)-(c): Approximate beam patterns for continuous-wave stimulation generated by the conjugate benchmark, the off-target suppression benchmark, and CORB, respectively. The color maps demonstrate the spatial distribution of mean ultrasound intensity normalized by the target signal intensity (0.078 W/cm^2). (d)-(f): Neuronal spike count map of conjugate benchmark, the off-target suppression benchmark, and CORB, respectively, in a continuous-wave LIFU stimulation (target intensity: 0.078 W/cm^2; duration: 100 ms). Ultrasound pressures at all other points are scaled proportionally to the target based on the mean intensity distributions in (a)-(c).

systems. The OTAAs in Fig. 6 and Fig. M-5 are comparable, while the continuous-wave stimulation elicits more spikes in both on-target and off-target regions. The same phenonmenon can be observed in Fig. 7 and Fig. M-6.

Appendix 1: Prove Formulation Equivalence of (M-3) And (M-4)

It is assumed that the optimal beamforming vector that minimize $S(w)$ is w_S^*. The intensity $I(w,G)$ is a function of w in (M-2) and is non-negative. If $I(w_S,G^*) > I_h$, then there exists $\sigma < 1$ such that $I_h = I(\sigma w_S, G^*) = \sigma^2 I(w_S, G^*) \leq I(w_S, G^*)$. This

inequality $I(\sigma w_S, G) \leq I(w_S, G)$ also holds for any other brain region G. Since $S(w)$ is an increasing function of $I(w, G)$, it can be concluded that $S(\sigma w_S) \leq S(w_S)$. Then, it can be inferred that either w_S is not optimal, contradicting the assumption, or σw_S is also an equivalently optimal beamforming vector. In either cases, the optimal beamforming vector can be equivalently solved when the tighter constraint $I(w, G^*) = I_h$ is satisfied.

Given the analysis above, it can always be assumed that $[w]^H Q(G^*) w = I_h$ while solving the optimization. Then by substituting $\frac{I_l}{I_h}[w]^H Q(G^*) w$ for I_l, (M-3) can be equivalently transformed to (M-4). The power of w does not affect the value of the objective function in (M-4).

Appendix 2: Logistic Function Steepness Parameter in (M-5)

This work derives a range of μ as a guideline for a specific scenario in which the existence of the "ideal beam pattern" is assumed. According to the requirement of high-resolution neuromodulation, an "ideal beam pattern" whose feasibility is not guaranteed should completely avoid off-target excitation with potential side effects. To simplify the notation, the objective function in (M-5) is represented by $F_2(\mu, w)$. The optimal beamforming vector that minimizes $F_2(\mu, w)$ given μ is defined as $w_2^*(\mu)$. If an ideal beam pattern w_S^* exists, based on the proved equivalence of w_S^* and w_1^*, $F_1(w_1^*) = 0$. The number of points in the brain tissue region, denoted by Ω_B, is defined as $|\Omega_B|$, which satisfies $|\Omega_B| \geq 3$ to perform non-trivial optimizations.

First, it is shown as follows that if there exists μ and an ideal beam pattern w_1^*, $w_2^*(\mu)$ is also an ideal beam pattern. As $F_1(w_1^*) = 0$, there exists a μ that is large enough such that $F_2(\mu, w_1^*) \leq \frac{1}{2}$ according to properties of the logistic function. On the other hand, $w_2^*(\mu)$ minimizes $F_2(\mu, w)$, which implies $F_2(\mu, w_2^*(\mu)) \leq F_2(\mu, w_1^*) \leq \frac{1}{2}$. Therefore, $[w_2^*(\mu)]^H Q^*(G) w_2^*(\mu) < 0$ for any point G according to (M-5), which implies $F_1(w_2^*(\mu)) = 0$. This means that $w_2^*(\mu)$ is also an optimal solution for (M-4) and an ideal beamforming vector given μ. Such a result validates the approximation in (M-5) and shows that the optimal beam pattern is preserved in this case.

It is preferred in optimization that (M-5) preserves the ideal beam pattern after approximation, and hence an appropriate value of μ is expected to ensure $F_2(\mu, w_1^*) \leq \frac{1}{2}$, as discussed above. Here, the approach is to derive a lower bound of $F_2(\mu, w_1^*)$ without solving for w_1^*, which represents the approximated area in (M-5) of an ideal beam pattern. As $F_1(w_1^*) = 0$, it can be inferred from (M-4) that $[w_1^*]^H Q^*(G) w_1^* < 0$ for any point G. As a logistic function is convex when the variables are negative, according to Jensen's inequality[45],

$$F_2(\mu, w_1^*) \geq \left(|\Omega_B| - 1\right)\left(1 + e^{-\mu [w_1^*]^H \bar{Q} w_1^*}\right)^{-1}, \tag{15}$$

where the average propagation difference matrix $\bar{Q}$ is

$$\bar{Q} = \frac{1}{|\Omega_B| - 1} \sum_G Q^*(G). \tag{16}$$

If Λ_{min} is further defined as the minimal eigenvalue of $\bar{Q}$, $[w_1^*]^H \bar{Q} w_1^* \geq \Lambda_{min} P$ when subject to the regularization power constraints in (M-4). Therefore,

$$F_2(\mu, w_1^*) \geq \left(|\Omega_B| - 1\right)\left(1 + e^{-\mu \Lambda_{min} P}\right)^{-1}, \tag{17}$$

which serves as a lower bound of the approximated area for any ideal beam pattern w_1^*.

Next, the lower bound is used to find a necessary range of μ such that $F_2(\mu, w_1^*) \leq \frac{1}{2}$. A necessary condition for the inequality is that the lower bound in (17) is not larger than $\frac{1}{2}$. Therefore, the necessary condition for μ is

$$\mu \geq \mu_{min} = -\frac{\ln(2|\Omega_B| - 3)}{\Lambda_{min} P}. \tag{18}$$

So far, a range of μ is derived as a guideline based on the assumption that the ideal beam pattern exists. When the optimal beam pattern w_1^* is not ideal, due to the existence of points with $[w_1^*]^H Q^*(G) w_1^* > 0$, Jensen's inequality cannot directly be used to derive (15). However, if $[w_1^*]^H \bar{Q} w_1^* < 0$ in (15), then based on properties of the logistic function, (15) can still be satisfied with a sufficiently large μ. Then (18) may still serve as a guideline for parameter selection.

References

1. Cocosco, C. A., Kollokian, V., Kwan, R. K.-S., Pike, G. B. & Evans, A. C. Brainweb: Online interface to a 3d mri simulated brain database. In *NeuroImage* (Citeseer, 1997).

2. Design and construction of a realistic digital brain phantom. *IEEE transactions on medical imaging* **17**, 463–468 (1998).

3. Rukundo, O. & Cao, H. Nearest neighbor value interpolation. *International Journal of Advanced Computer Science and Applications* **3**, 25–30 (2012).

4. Roitner, H., Bauer-Marschallinger, J., Berer, T. & Burgholzer, P. Experimental evaluation of time domain models for ultrasound attenuation losses in photoacoustic imaging. *The Journal of the Acoustical Society of America* **131**, 3763–3774 (2012).

5. Treeby, B. E., Jaros, J., Rendell, A. P. & Cox, B. Modeling nonlinear ultrasound propagation in heterogeneous media with power law absorption using a k-space pseudospectral method. *The Journal of the Acoustical Society of America* **131**, 4324–4336 (2012).

6. Mueller, J. K., Ai, L., Bansal, P. & Legon, W. Numerical evaluation of the skull for human neuromodulation with transcranial focused ultrasound. *Journal of neural engineering* **14**, 066012 (2017).

7. Deffieux, T. & Konofagou, E. E. Numerical study of a simple transcranial focused ultrasound system applied to blood-brain barrier opening. *IEEE transactions on ultrasonics, ferroelectrics, and frequency control* **57**, 2637–2653 (2010).

8. Mueller, J. K., Ai, L., Bansal, P. & Legon, W. Computational exploration of wave propagation and heating from transcranial focused ultrasound for neuromodulation. *Journal of neural engineering* **13**, 056002 (2016).

9. Hasgall, P. *et al.* It'is database for thermal and electromagnetic parameters of biological tissues. version 4.0, may 15, 2018. doi: 10.13099 (2018).

10. Scherba, E., Hoagland, P. A. & O'Brien, W. D. Acoustic microscopy: a study of contrast in fresh tissue. *IEEE transactions on ultrasonics, ferroelectrics, and frequency control* **41**, 451–457 (1994).

11. Selbekk, T. *et al.* Ultrasound imaging in neurosurgery: approaches to minimize surgically induced image artefacts for improved resection control. *Acta neurochirurgica* **155**, 973–980 (2013).

12. Kremkau, F. W., Barnes, R. W. & McGraw, C. P. Ultrasonic attenuation and propagation speed in normal human brain. *The Journal of the Acoustical Society of America* **70**, 29–38 (1981).

13. Azhari, H. *Basics of biomedical ultrasound for engineers* (John Wiley & Sons, 2010).

14. Kyriakou, A., Neufeld, E., Werner, B., Székely, G. & Kuster, N. Full-wave acoustic and thermal modeling of transcranial ultrasound propagation and investigation of skull-induced aberration correction techniques: a feasibility study. *Journal of therapeutic ultrasound* **3**, 1–18 (2015).

15. White, P. J., Clement, G. T. & Hynynen, K. Longitudinal and shear mode ultrasound propagation in human skull bone. *Ultrasound in medicine & biology* **32**, 1085–1096 (2006).

16. Law, J. & Rennie, R. *A dictionary of physics* (OUP Oxford, 2015).

17. Vallero, D. *Air pollution calculations: Quantifying pollutant formation, transport, transformation, fate and risks* (Elsevier, 2019).

18. Jakevičius, L. & Demčenko, A. Ultrasound attenuation dependence on air temperature in closed chambers. *Ultragarsas (Ultrasound)* **63**, 18–22 (2008).

19. Legon, W. *et al.* Transcranial focused ultrasound modulates the activity of primary somatosensory cortex in humans. *Nature neuroscience* **17**, 322–329 (2014).

20. Treeby, B. E. & Cox, B. T. Modeling power law absorption and dispersion for acoustic propagation using the fractional laplacian. *The Journal of the Acoustical Society of America* **127**, 2741–2748 (2010).

21. Treeby, B. E. & Cox, B. T. Modeling power law absorption and dispersion in viscoelastic solids using a split-field and the fractional laplacian. *The Journal of the Acoustical Society of America* **136**, 1499–1510 (2014).

22. He, Z., Shen, Y. & Wang, Q. Boundary extension for hilbert–huang transform inspired by gray prediction model. *Signal Processing* **92**, 685–697 (2012).

23. Pasquinelli, C. *et al.* Transducer modeling for accurate acoustic simulations of transcranial focused ultrasound stimulation. *Journal of neural engineering* **17**, 046010 (2020).

24. Chaplin, V., Phipps, M. A. & Caskey, C. F. A random phased-array for mr-guided transcranial ultrasound neuromodulation in non-human primates. *Physics in Medicine & Biology* **63**, 105016 (2018).

25. Jiang, X. *et al.* A review of low-intensity pulsed ultrasound for therapeutic applications. *IEEE Transactions on Biomedical Engineering* **66**, 2704–2718 (2018).

26. Gougheri, H. S., Dangi, A., Kothapalli, S.-R. & Kiani, M. A comprehensive study of ultrasound transducer characteristics in microscopic ultrasound neuromodulation. *IEEE transactions on biomedical circuits and systems* **13**, 835–847 (2019).

27. Seok, C., Ali, Z., Yamaner, F. Y., Sahin, M. & Oralkan, Ö. Towards an untethered ultrasound beamforming system for brain stimulation in behaving animals. In *2018 40th Annual International Conference of the IEEE Engineering in Medicine and Biology Society (EMBC)*, 1596–1599 (2018).

28. Kim, S. *et al.* Transcranial focused ultrasound stimulation with high spatial resolution. *Brain Stimulation* **14**, 290–300 (2021).

29. Jones, R. M., Caskey, C. F., Dayton, P. A., Oralkan, Ö. & Pinton, G. F. Transcranial neuromodulation array with imaging aperture for simultaneous multifocus stimulation in nonhuman primates. *IEEE Transactions on Ultrasonics, Ferroelectrics, and Frequency Control* **69**, 261–272 (2021).

30. Thomas, J.-L. & Fink, M. A. Ultrasonic beam focusing through tissue inhomogeneities with a time reversal mirror: application to transskull therapy. *IEEE transactions on ultrasonics, ferroelectrics, and frequency control* **43**, 1122–1129 (1996).

31. He, Z. *et al.* A sidelobe suppressing near-field beamforming approach for ultrasound array imaging. *The Journal of the Acoustical Society of America* **137**, 2785–2790 (2015).

32. Hertzberg, Y., Naor, O., Volovick, A. & Shoham, S. Towards multifocal ultrasonic neural stimulation: pattern generation algorithms. *Journal of neural engineering* **7**, 056002 (2010).

33. Naor, O., Hertzberg, Y., Zemel, E., Kimmel, E. & Shoham, S. Towards multifocal ultrasonic neural stimulation ii: design considerations for an acoustic retinal prosthesis. *Journal of neural engineering* **9**, 026006 (2012).

34. Iero, D., Isernia, T. & Crocco, L. Focusing time harmonic scalar fields in non-homogenous lossy media: inverse filter vs. constrained power focusing optimization. *Applied Physics Letters* **103**, 093702 (2013).

35. Huang, Y., Datta, A. & Parra, L. C. Optimization of interferential stimulation of the human brain with electrode arrays. *Journal of neural engineering* **17**, 036023 (2020).

36. Kamimura, H. A. *et al.* Focused ultrasound neuromodulation of cortical and subcortical brain structures using 1.9 mhz. *Medical physics* **43**, 5730–5735 (2016).

37. Treeby, B. E. & Cox, B. T. k-wave: Matlab toolbox for the simulation and reconstruction of photoacoustic wave fields. *Journal of biomedical optics* **15**, 021314 (2010).

38. Azar, L., Shi, Y. & Wooh, S.-C. Beam focusing behavior of linear phased arrays. *NDT & e International* **33**, 189–198 (2000).

39. Foote, K. G. Discriminating between the nearfield and the farfield of acoustic transducers. *The Journal of the Acoustical Society of America* **136**, 1511–1517 (2014).

40. Corless, R. M., Gonnet, G. H., Hare, D. E., Jeffrey, D. J. & Knuth, D. E. On the lambert w function. *Advances in Computational mathematics* **5**, 329–359 (1996).

41. Cox, H., Zeskind, R. & Owen, M. Robust adaptive beamforming. *IEEE Transactions on Acoustics, Speech, and Signal Processing* **35**, 1365–1376 (1987).

42. Zhang, Y., Ko, Y., Woods, R. & Marshall, A. Defining spatial secrecy outage probability for exposure region-based beamforming. *IEEE Transactions on Wireless Communications* **16**, 900–912 (2016).

43. Turner, L. R. Inverse of the vandermonde matrix with applications (1966).

44. Campbell, R. (2020). URL `https://www.github.com/raacampbell/shadedErrorBar`.

45. Mercer, A. M. A variant of jensen's inequality. *Journal of Inequalities in Pure and Applied Mathematics* **4** (2003).
